# Assessment of root and root canal morphology in maxillary molars with fused roots using Cone Beam Computer Tomography (CBCT) in a Sri Lankan population

**DOI:** 10.1016/j.jobcr.2025.08.016

**Published:** 2025-08-19

**Authors:** Ruvienath Daham Weerasinghe Rajapaksa, Manil Christopher Nishan Fonseka, Ruwan Duminda Jayasinghe, Rasika Manori Jayasinghe

**Affiliations:** a Restorative Dentistry, Postgraduate Institute of Medicine, University of Colombo, Sri Lanka; b Restorative Dentistry, Department of Restorative Dentistry, Faculty of Dental Sciences, University of Peradeniya, Sri Lanka; c Oral Medicine and Periodontology, Department of Oral Medicine and Periodontology, Faculty of Dental Sciences, University of Peradeniya, Sri Lanka; d Prosthetic Dentistry, Department of Prosthetic Dentistry, Faculty of Dental Sciences, University of Peradeniya, Sri Lanka

## Abstract

**Introduction:**

Root fusion is considered to present when there is no evidence of periodontal space or presence of bone between the different roots of the molar at any apical level to the bifurcation area. Fused roots in maxillary molars pose important clinical implications, mainly in the field of endodontics. Based on the wide variations in previous studies done in different populations and the clinical implications, the present study is aimed to assess root and root canal morphology in maxillary first and second molars with fused roots in a Sri Lankan population.

**Material and methods:**

A descriptive study was conducted by evaluating all CBCT scans stored at Division of Oral Medicine and Radiology, Faculty of Dental sciences, University of Peradeniya which were taken from January 1st, 2017 to December 31st, 2019. To characterize the type of root fusion of maxillary molars, classification of Zhang et al., in 2014 was used.

**Results:**

Out of one thousand twenty upper first molars (1020), fifty two had fused roots (5.098 %) and out of one thousand ninety-six upper second molars (1096), 473 (43.15 %) had fused roots. The commonest pattern of fusion noted in first molars was type 1 (42.3 %) and in second molars was type 2 (36.9 %).

**Conclusion:**

The root and canal configurations of maxillary first and second molars in this population were consistent with previously reported data. Fused roots may present a complicated root canal system. These data may facilitate successful endodontic treatment. More studies in larger populations would provide more details in our population.

## Graphical abstract

**Figure:**
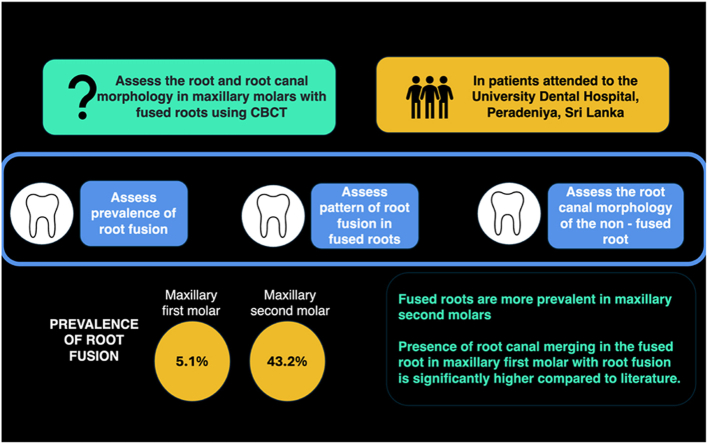

## 1 Background

The term ‘fused root’ denotes the union of two or more roots, which may occur either through the deposition of hard tissues during the individual's lifetime or due to developmental alterations in Hertwig's epithelial root sheath within the furcation region.^1^^,^^2^ Fused roots commonly occur among maxillary molars, particularly in maxillary second molars and they can also occur in mandibular molars.^3^

To date multiple studies have been conducted regarding assessment of root and root canal morphology of fused roots and a wide variation in results have been noted. CBCT studies done in an Asian population has yielded much higher prevalence as 19.5 % in maxillary first molars and 46.5 % in maxillary second molars.^4^ Few recent CBCT studies of different populations have shown varying results. In the Turkish population, the incidence of fused roots in the maxillary first molars and maxillary second molars was 7.14 % and 23.47 %, respectively, with more canal merging in the maxillary second molars.^5^ In the Saudi Arabian population maxillary second molars presented a more complex external and internal morphology compared with maxillary first molars, with an overall prevalence of 14 % of fused rooted maxillary molars and 3.7 % merged and 0.8 % C-shaped canal configurations for all maxillary molars.^6^

Knowledge of the root and root canal morphology of fused roots is pertinent for various aspects of clinical dental practice.^7^ Fused roots present significant challenges from endodontic, periodontal, and endodontic microsurgical perspectives.^8^ Molars exhibiting root fusion often demonstrate substantial anatomical variations in root and root canal morphology, posing challenges in the identification, cleaning, shaping, and obturation of the root canal system.^9^ Therefore, the clinicians should be aware of the possible variations in order to avoid complications along the course of treatment sessions.

In addition, a depression can be formed at the root surface due to the fused roots which allow bacterial migration leading to periodontal attachment loss and subsequent bone loss.^10^^,^^11^ In a study evaluating root canal morphology of maxillary and mandibular second molars lost due to periodontitis, Kato et al. reported a higher frequency of root fusion in maxillary and mandibular second molars lost due to periodontitis.^12^ Furthermore, dentine wall adjacent to these grooves can be thin and more susceptible to perforations during canal shaping in endodontic procedure.^13^

In the past various techniques have been employed to study root and root canal morphology, including staining and clearing methods, conventional radiography, spiral computed tomography (CT), contrast-enhanced radiography, and more recently CBCT. Tooth cleaning and staining methods have been considered gold standard.^14^ Peiris R in 2008 used clearing technique to analyze root canal morphology of human permanent dentition in a group of Sri Lankan population.^15^ However as clearing techniques are ex vivo studies conducted in extracted teeth and are highly useful for visualizing canal morphology in detail and there are some inherent drawbacks such as inability to fully replicate the in vivo state and labor – intensive and has been largely replaced by micro computed tomography in modern research. Micro-computed tomography (micro-CT) is an X-ray imaging technique that produces high-resolution, 3D images of small objects. it is a powerful tool for research, particularly in preclinical and experimental studies. It offers high-resolution, 3D imaging of small specimens, making it useful for examining tissue structure. However, micro-CT is not a standard clinical imaging technique for patients due to long scan times, mechanical stability requirements and limited penetration. All in vitro methods, such as the tooth-clearing technique or micro-CT, are limited to use on extracted teeth, and therefore unable to detect bilateral and adjacent tooth morphology and restricts their applicability in clinical practice.

In clinical practice, conventional radiographs such as periapical radiographs provide 2D visualization of a 3D structure, therefore are subjected to distortion and superimposition. Cone-beam CT enables three-dimensional visualization of root and root canal morphology, offering a lower radiation dose and higher spatial resolution compared to conventional CT.^16^ CBCT allows the teeth to be examined in the in vivo state and allows bilateral comparison of teeth. However, CBCT has limitations in terms of spatial resolution and radiation exposure, which may not make it ideal for detailed canal morphology analysis.

Only a few studies have been conducted to assess root fusions patterns in maxillary molars using CBCT and they have yielded wide variation in results in different populations. Only a single study has been done to date to assess the prevalence of root fusion in a Sri Lankan population, which was conducted on extracted teeth using clearing and staining technique.^15^ However, the variation in prevalence of root fusion in relation to sex and ethnicities was not elaborated this study. Furthermore, hardly any study has assessed the root canal morphology in fused maxillary molars in Sri Lanka.

Based on the wide variations in previous studies done in different populations and the clinical implications, the present study is aimed to assess root and root canal morphology in maxillary first and second molars with fused roots in a Sri Lankan population. It would enhance success of outcome in dental treatment for patients in the country.

## 2 Materials and methods

This descriptive cross-sectional study was approved by the Ethics Committee of Faculty of Dental Sciences, University of Peradeniya. The study was conducted in the Division of Radiology, Department of Oral Medicine and Periodontology, Faculty of Dental Sciences, University of Peradeniya over a period of one year from January 1, 2020 to December 31, 2020.

A convenient sampling method was employed where all CBCT scans which were taken various reasons such as assessment of maxillary and mandibular pathology, orthodontic reasons and implant planning were taken into consideration. CBCT scans which support inclusion and exclusion criteria and stored at Division of Radiology, Department of Oral Medicine and Periodontology, Faculty of Dental Sciences, University of Peradeniya from January 1st, 2017 to December 31st, 2019, were assessed for the purpose of this study. CBCT scans with fully erupted maxillary first and second molars with closed apex and with bilateral maxillary first and second molars were included for the purpose of this study. As teeth with normal anatomy are considered in this study, teeth with pathological conditions affecting the roots and root canal system such as teeth with root resorption, root caries, periapical pathologies, teeth affected by developmental anomalies and teeth with calcified canals are excluded. Endodontically treated teeth are excluded because the root canal anatomy may have changed during canal preparation.

Pax - Duo3D version 4.1.0 CBCT machine was used in this study and CBCT software (EZ3D Plus) was used to view the CBCTs. CBCT scans of adequate diagnostic quality void of artefacts and showing bilateral fully erupted molars with closed apex and devoid of periapical pathologies were be included in the study. The age, sex and ethnicity of subjects were be identified from the records and the side of the molar teeth will be recorded.

The acquired scans were converted to digital imaging and communications in medicine (DICOM) format and were reconstructed into multi planar reconstruction images using the DICOM viewer and were assessed in axial, coronal, sagittal, trans axial and pseudo OPG views with the minimum available thickness.

### 2.1 Assessment of fused root/s of fused molar teeth

**Fig. 1:**
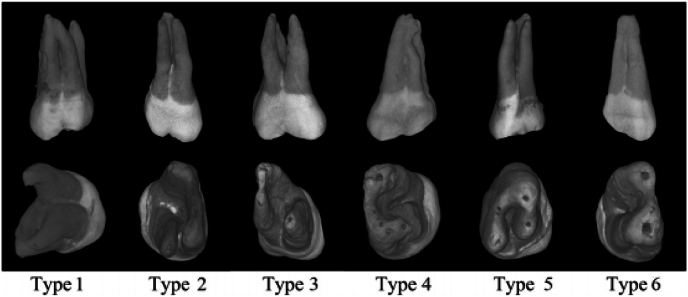
Type of root fusion of maxillary molars, classification of Zhang et al., 2014. Type 1: MBR fused with DB, Type 2: MBR fused with PR, Type 3: DBR fused with PR, Type 4: MBR fused with DBR and PR or PR with MBR and DBR (proximal groove), Type 5: PR fused with MBR and DBR, Type 6: PR, MBR, and DBR fused to a cone-shaped root” MBR- Mesiobuccal root, DBR- Distobuccal root, PR- Palatal root.

Root fusion was considered to present when there is no evidence of periodontal space or presence of bone between the different roots of the molar at any apical level to the bifurcation area, according to the criteria proposed by Ross and Evanchik in 1981(3). To characterize the type of root fusion of maxillary molars, classification of Zhang et al., in 2014 was used.^17^ (Fig. 1).

**Fig. 2:**
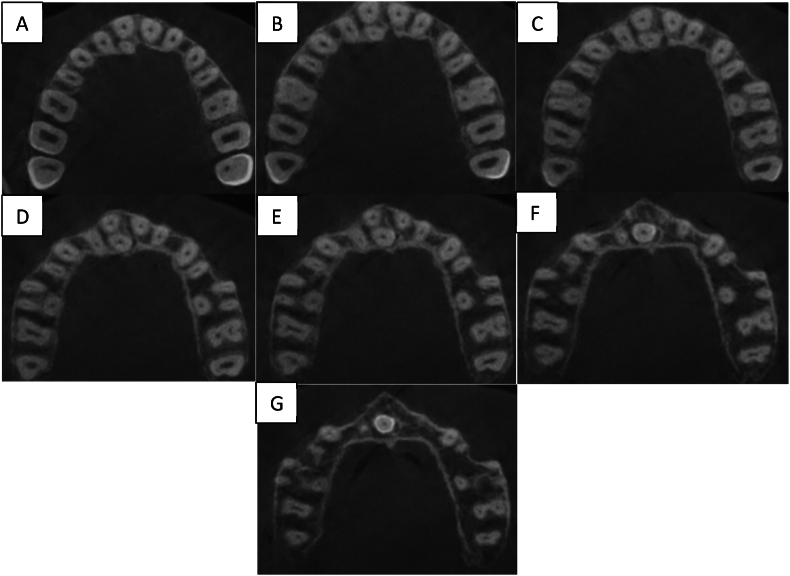
– A to G Axial sections showing Type 6 in Right MSM and Type 4 in Left MSM.

Axial views along the roots were assessed to determine the type of root fusion according to the classification of Zhang et al. (Fig. 2)

Fused roots were further analyzed to assess the number of canals, whether the canals merge or not and their location of merging (coronal, middle or apical 1/3rd).

### 2.2 Assessment of non-fused roots in fused molar teeth

**Fig. 3:**
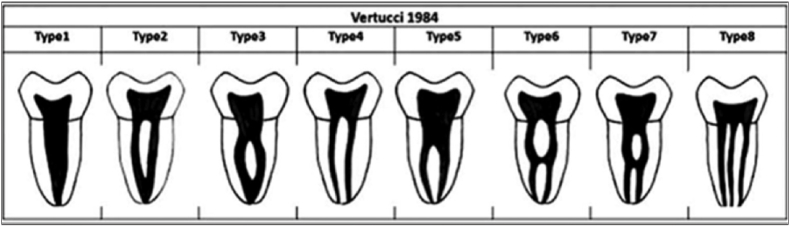
Vertucci's classification of root canal morphology.

Trans axial view was used to assess the root canal morphology of the maxillary molars and the non-fused root of the fused molar was classified according to the Vertucci's classification.^7^ (Fig. 3).

The findings were noted down for maxillary first and second molars separately in data collection sheets.

The training and calibration were done by an expert in Radiology (RDJ) using randomly selected 50 CBCTs. The observer (RR) received a detailed explanation regarding the morphological features root canal system using the Zhang et al., 2014 classification for fused roots and Vertucci classification for non-fused roots using a PowerPoint presentation containing high resolution images.

Calibration was performed twice, with a four-week washout period between sessions. The recorded data were analyzed to assess inter-examiner agreement, and the observer was subsequently asked to re-evaluate the slices to identify any intra-examiner variability.

## 3 Statistical analysis

Patient's age, sex, ethnicity, root canal morphology of fused root/s according to Zhang et al., 2014 classification, Presence or absence of merging, and if present, location of merging of fused root and morphology of the non-fused root of fused molar according to the Vertucci classification, were tabulated. Weighted kappa coefficients were calculated using STATA version 16 to evaluate intra- and inter-examiner measurement reliability, with results interpreted according to the Landis and Koch classification scale.^18^ Statistical analysis was done using SPSS version 24 (SPSS inc., Armonk, NY, USA) software for windows.

Fisher's exact probability test was used to assess the statistical significance between sex and type of fusion of maxillary first molars, and ethnicity and type of fusion of maxillary first molars. Pearson's Chi-squared test was used to assess the statistical significance between sex and type of fusion of maxillary second molars, ethnicity and type of fusion of maxillary second molars and type of fusion between first and second maxillary molars.

## 4 Results

A total of 2116 teeth of 560 subjects were assessed out of which 1020 were maxillary first molars and 1096 were maxillary second molars. 52 out of 1020 maxillary first molars (MFM) had fused roots resulting in a prevalence of 5.098 % and 473 maxillary second molars (MSM) out of 1096 had fused roots resulting in a prevalence of 43.15 %. Overall prevalence of root fusion in maxillary molars were 24.81 %. The weighted kappa coefficients for assessing intra- and inter-examiner measurement error were 0.80 and 0.82, respectively, indicating an almost perfect level of agreement based on the Landis and Koch scale.^18^

For ease of data presentation and analysis the subjects with molars with root fusion were grouped according to their age in the following manner.

G1 16–25 years
G2 26–35 years
G3 36–45 years
G4 46–55 years

**Fig. 4:**
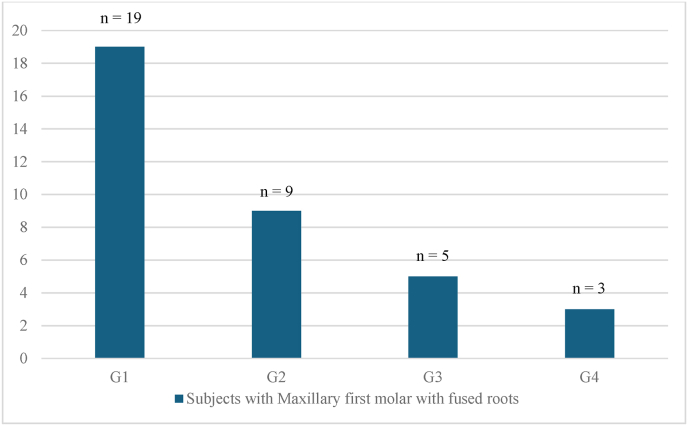
– Age distribution of subjects with maxillary first molars with fused roots G1:16–25 years, G2: 26–35 years, G3: 36–45 years, G4: 46–55 years.

**Fig. 5:**
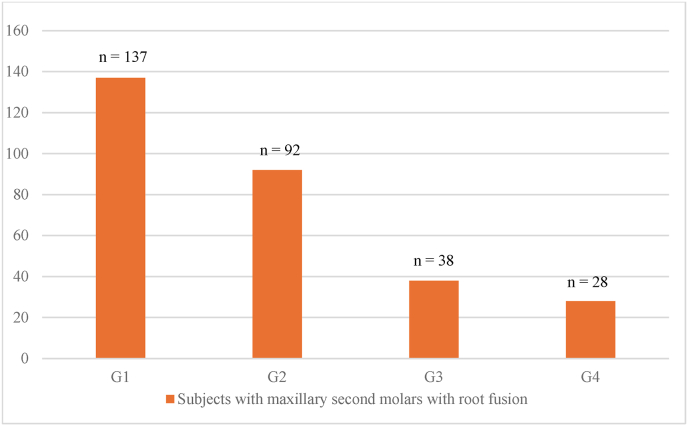
Age distribution among subjects with maxillary second molars with fused roots G1:16–25 years, G2: 26–35 years, G3: 36–45 years, G4: 46–55 years.

Age distribution among the subjects with MFM and MSM molars with root fusion followed a similar pattern, with age group G1 (16–25 years) predominant in both. (Fig. 4, Fig. 5).

Females had a higher prevalence of root fusion (60.4 %) compared to males (39.7 %). Sex distribution was similar between subjects with MFM with fused roots and MSM with fused roots as well and female preponderance was noted in both (Table 1).

**Table 1:** Sex distribution among subjects with maxillary first and second molars with fused roots.

Sex	MFM		MSM	
	n	%	n	%
Male	11	30.5	108	36.6
Female	25	69.4	187	63.4

MFM – Maxillary first molar.

MSM – Maxillary second molar.

Sinhala was the most prevalent ethnic group among subjects with fused roots in MFM (88.9 %) followed by Tamil (5.6 %), Muslim (2.8 %) and Other (2.8 %). Sinhala was the most prevalent ethnic group in MSM with fused roots as well (90.5 %) followed by Muslim (5.1 %), Tamil (4.1 %) (Table 2). Prevalence of root fusion was most prevalent among the Sinhala ethnic group (52.9 %), followed by Tamil (48.2 %) and Muslim (31.3 %)

**Table 2:** Distribution of ethnicities among subjects with maxillary first and second molars with fused roots.

Ethnicity	MFM		MSM	
	n	%	n	%
Sinhala	32	88.9	267	90.5
Tamil	2	5.6	12	4.1
Muslim	1	2.8	15	5.1
Other	1	2.8	2	0.4

MFM – Maxillary first molar.

MSM – Maxillary second molar.

### 4.1 Analysis of the fused roots in MFM and MSM with fused roots

**Fig. 6:**
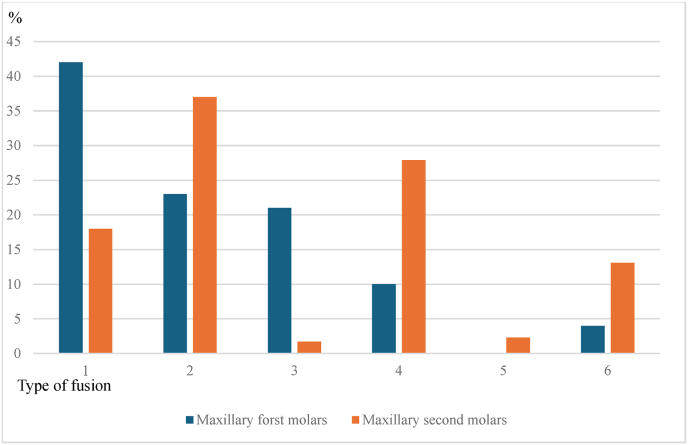
Percentage distribution of types of fusion in maxillary first and second molars with fused roots.

**Fig. 7:**
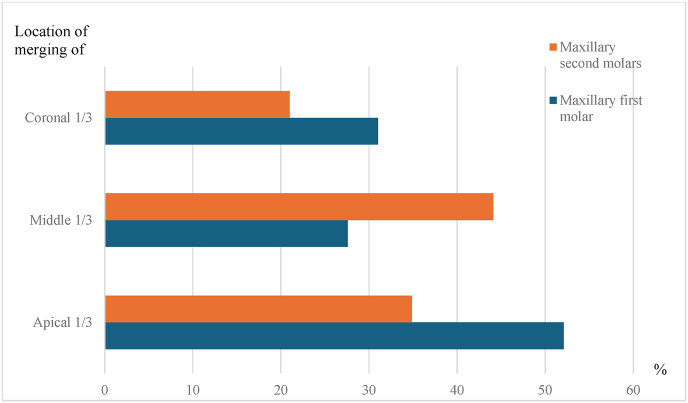
Percentage distribution of the location of root canal merging in fused roots in maxillary first and second molars.

In MFM with fused roots, the most prevalent type of fusion observes was Type 1(42 %), followed by Type 2 (23 %), Type 3 (21 %) and Type 6 (0.04 %). Type 5 fusion pattern was not observed in the sample (Table 3 and Fig. 6).(see Fig. 7).

**Table 3:** Distribution of type of fusion in the fused root in maxillary first molar teeth.

Type of fusion	Male (M)		Female (F)		M + F	
	n	%	n	%	n	%
1	3	6.0	19	37.0	22	42.0
2	5	10.0	7	13.0	12	23.0
3	5	10.0	6	12.0	11	21.0
4	0	0.0	5	5.0	5	10.0
5	0	0.0	0	0.0	0	0.0
6	2	4.0	0	0.0	2	4.0

**Table 4:** Distribution of type of fusion in the fused root in maxillary second molar teeth.

Type of fusion	Male (M)		Female (F)		M + F	
	n	%	n	%	n	%
1	37	7.8	48	10.1	85	18.0
2	70	14.8	105	22.2	175	37.0
3	5	1.1	3	0.6	8	1.7
4	36	7.6	96	20.3	132	27.9
5	5	1.1	6	1.3	11	2.3
6	19	4.0	43	9.1	62	13.1

In males, the commonest type of root fusion observed were types 2 and 3 (33.3 % each) while in females the commonest pattern of fusion observed was type 1 (51.35 %) (see Table 4).

Statistical significance between the two sexes with respect to type of fusion was assessed using Fisher's exact probability test as Chi-squared test cannot be applied as the counts of 5 cells are less than 5. Since the p-value = 0.0168 < 0.05, there is enough evidence to state that the null hypothesis is rejected at 5 % significance level and there is a statistically significant relationship between the two sexes and type of fusion in MFM.

Similarly, Fisher's exact probability test was used to assess the statistical significance between ethnicity and type of fusion. Since the p-value = 0.7244 > 0.05, there is enough evidence to say that the null hypothesis is not rejected at 5 % significance level, i.e. There is no association between the two variables, the Ethnicity and Type of fusion.

In contrast, in MSM with fused roots, the most prevalent pattern of fusion detected was Type 2 (37 %) followed by Type 4 (27.9 %), Type 1(18) and Type 6 (13.1 %). Type 5 was seen in 11 (2.3 %) of the subjects and the least prevalent pattern was Type 3 (1.7 %) (Table 3 and Fig. 5). In males, the most prevalent pattern of root fusion was type 2 (40.69 %) followed by type 1 (21.51 %) and type 4 (20.93 %). Among females, the commonest type of root fusion observed was type 2 (34.88 %) followed by type 4 (31.89 %) and type 1(15.94 %).

Statistical significance between the two sexes with respect to type of fusion was assessed using Pearson's Chi-squared test. Since the p-value = 0.03548 < 0.05, there is enough evidence to highlight that the null hypothesis is rejected at 5 % significance level, and therefore, there is an association between Sex and Type of Fusion in MSM.

Fisher's exact probability test was used to assess the statistical significance between ethnicity and type of fusion. Since the p-value = 0.7954 > 0.05, there is enough evidence to say that the null hypothesis is not rejected at 5 % significance level, i.e. There is no association between the two variables the Ethnicity and Type of fusion.

Pearson's Chi-squared test was used to assess the association between the two variables Molar Type and Type of Fusion Since the p-value = 0.0004998 < 0.05, there is enough evidence to say that the null hypothesis: is rejected at 5 % significance level, i.e. there is statistically significant relationship between the type of molar and the type of fusion (see Table 5).

Merging of the root canal in the fused roots was observed in 56 % of the MFM. Out of the teeth with merging root canals 52.17 % was in apical 1/3, 27.59 % was in middle 1/3 and 31.03 % was in coronal 1/3. In MSM, merging of root canals in the fused root was observed in 41.5 % teeth. Location of merging observed in the teeth with fused roots was middle 1/3 44.1 %, coronal 1/3 34.87 % and apical 1/3 in 21 % (Table 6, Table 7 and Fig. 6).

**Table 5:** Distribution of type of fusion in the fused root between maxillary first and second molar teeth.

Type of fusion	MFM		MSM	
	n	%	n	%
1	22	42.0	85	18.0
2	12	23.0	175	37.0
3	11	21.0	8	2.0
4	5	10.0	132	28.0
5	0	0.0	11	2.0
6	2	4.0	62	13.0

MFM – Maxillary first molar.

MSM – Maxillary second molar.

**Table 6:** Presence of merging in the fused root in maxillary first and second molar teeth.

Root canal merging/not	MFM		MSM	
	n	%	n	%
Merging	29	56.0	197	41.6
Not merging	23	44.0	278	58.4

**Table 7:** Location of merging in the fused root in maxillary first and second molar teeth.

	MFM		MSM	
	n	%	n	%
Apical 1/3	12	52.10	41	21.00
Middle1/3	8	27.59	86	44.10
Coronal 1/3	9	31.03	68	34.87

### 4.2 Analysis of the non-fused root in MFM and SFM with fused roots

In both MFM and MSM, most prevalent root canal morphology observed in the non – fused root was type V1. The prevalence of the type of root canal morphology is summarized in Table 8.

**Table 8:** Root canal morphology in the non-fused root in MFM and MSM.

Root canal morphology in non-fused root	MFM		MSM	
	n	%	n	%
V1	28	54.0	228	48.2
V2	12	23.0	33	7.0
V3	2	4.0	4	0.8
V4	2	4.0	0	0.0
V5	1	2.0	1	0.2
V6	0	0.0	0	0.0
V7	0	0.0	1	0.2
V8	0	0.0	0	0.0

MFM – Maxillary first molar.

MSM – Maxillary second molar.

## 5 Discussion

The morphology of the root canal system is a crucial factor in the success of endodontic treatment. Improper identification of root canal anatomy can result in inadequate treatment leading to disastrous outcomes such as treatment failure and complications such as missed canals. Variations in root and canal anatomy, particularly in maxillary molars, can pose significant challenges for clinicians during root canal treatment procedures.^19^) Maxillary molars are known to exhibit a wide range of anatomical configurations, including the presence of additional roots and root canals.^20^^,^^21^ Knowledge of the root and root canal morphology of fused roots is pertinent for various aspects of clinical dental practice as it can pose a challenge for endodontic, periodontal and endodontic microsurgery point of view.^7^^,^^8^ The presence of fused roots in maxillary molars can further complicate the identification of individual root canals, making it essential to utilize advanced imaging techniques to accurately assess the root canal configuration.^21^

To date, several studies investigating the root and root canal morphology using CBCT have been conducted in various populations yielding a wide variation in results.^1^^,^^4^^,^^5^^,^^8^^,^^17^^,^^3^, ^22^, ^23^, ^24^ To the best of our knowledge, this is the first study to assess the root and root canal morphology in maxillary molars with fused roots using CBCT in a Sri Lankan population.

A wide range of overall prevalence of fusion in maxillary molars ranging from 14 - 43 % has been reported in similar studies done in different populations. The prevalence of root fusion in maxillary molars in our study was 24.82 %.

Similarly, a wide variation of the prevalence of root fusion in MFM was also among the studies. Prevalence as low as 0.73 % was reported by Kim et al., 2012^14^ and prevalence as high as 23.6 % was observed in a study conducted by Marcano – Caldera et al., 2019.^8^ In our study prevalence of root fusion in MFM was reported as 5.1 %.

Similarly, a wide variation in prevalence of root fusion of MSM was also noted in literature, from 7.6 %^3^ to 57.6 %.^8^ In our sample the prevalence reported was 43.2 % which was towards the higher end of the range.

Peiris et al., 2008 reported 1.4 % and 47.5 % prevalence of root fusion in MFM and MSM respectively in a Sri Lankan population using clearing and staining technique on extracted teeth. In comparison, the present study reported higher prevalence if root fusion in MFM.

Multiple studies reported that the fusion between the palatal root and distobuccal root was the commonest form of fusion present in MFMs. In contrast, Zheng et al.^4^ reported fusion between mesiobuccal and distobuccal roots were the commonest pattern of fusion in MFMs. Prevalence of fusion between mesiobuccal and distobuccal root was reported as 42 % in our study and our results were similar to that of Zheng et al.^4^

In MSMs, the commonest pattern of fusion reported varied among the studies. Fusion between the mesiobuccal and palatal root was the commonest type of fusion (37 %) reported in the present study which coincided with the results of several studies.^5^^,^^6^ To the contrary, few studies reported commonest type of fusion to be between mesiobuccal and distobuccal roots^17^ or all three roots.^8^ All three micro – CT studies conducted on maxillary molars with root fusion reported also reported that the commonest type of fusion is between mesiobuccal and distobuccal roots.^1^^,^^17^^,^^25^ The relationship between the pattern of fusion and the type of molar was found to be statistically significant in our study similar to Marcano – Caldera et al.^8^ Our study observed a Our study observed a higher prevalence of root fusion among females similar to Martins et al. and a statistically significant relationship between sex and the type of fusion with respect to both MFM and MSMs. These results were similar to that of Marcano – Caldera et al.^2^^,^^8^

To the best of our knowledge this study if the first to assess prevalence of root fusion in different ethnic groups in Sri Lanka. Higher prevalence of root fusion was observed in Sinhala and Tamil ethnic groups (52.9& and 48.2 % respectively). Clinicians should maintain a heightened awareness of potential root fusion when managing patients from these ethnic groups.

Bilateral occurrence of root fusion in MFM and MSM reported in the present study (0.7 %) was significantly lower than reported by similar studies such as Mashyakhy et al. which reported approximately bilateral occurrence of root fusion in 3.8 % MFMs 13 % MSMs.^6^

Regarding the presence of fused roots in maxillary first and second molars in the same patient, in the present study, we found this in only 43 subjects of 560, representing 7.6 % of the total number of patients. 43 out of 51 patients with root fusion of MFM had a MSM with root fusion. Therefore, clinicians should be aware when a maxillary first molar tooth presents with fused roots, there is a 84.3 % chance of finding a maxillary second molar with root fusion which is a significant proportion.

Merging of the root canal in fused roots was not frequently assessed in many of the studies. In literature, the prevalence of root canal merging in fused roots of MFM were reported as 8.3 % and 18.2 %.^5^^,^^6^ Comparatively, the prevalence of root canal merging in the fused roots of MFMs (56 %) was significantly higher in our study.

In contrast, the prevalence of root canal merging in fused roots of MSM was reported as 41.6 % in the present study which was within the range reported in literature.^5^^,^^6^^,^^17^

Location of merging of root canal in the fused roots was assessed only in a single study which reported the most prevalent location for root canal merging in the fused root of MSM was middle 1/3 (43.75 %) which was similar to the present study.^17^ Root canal anatomy of maxillary molars with root fusion was not specifically discussed in the study by Peiris et al. which was done in a Sri Lankan population and used clearing and staining technique.^15^ Anatomy of the root canal system of maxillary molars with root fusion was explained in detail in micro-CT studies and has reported higher incidence of merging canals, apical deltas and isthmuses at apical level.^1^^,^^17^^,^^25^ Kato et al., 2021 also reported significant relationship between the number of root canals and the depth of root concavity in the mesiobuccal root of the maxillary first molars which has significant implications in periodontal treatment. However, due to the limitations in the spatial resolution in CBCT imaging fine details such as apical deltas, isthmuses and root concavities were not assessed in the present study.

As summarized in Table 9, previous studies have reported a wide range of variations in the root and root canal morphology of maxillary molars with root fusion across different populations. These variations can be attributed to genetic, racial, and geographical factors. By examining the root and root canal morphology of maxillary molars with fused roots in a Sri Lankan population, this study aims to contribute to the existing knowledge on the diversity of root and root canal anatomy and provide clinicians with valuable information to improve the success of clinical treatment specifically, endodontic treatment.

**Table 9:** Summary of studies assessing maxillary molars with root fusion using CBCT.

Author	Studied population	Prevalence of root fusion in MFM	Prevalence of root fusion in MSM	Overall prevalence of root fusion in maxillary molars	Most prevalent type of fusion in MFM	Most prevalent type of fusion in MSM	Prevalence of merging in MFM	Prevalence of merging in MSM
Present study	Sri Lankan	5.1 %	43.2 %	24.82 %	MBR + DBR	MBR + PR	56.0 %	41.6 %
Aydin et al., 2021(5)	Turkish population	7.14 %	23.47 %	N/A	DBR + PR	MBR + PR	18.2 %	40.6 %
Guo et al., 2014^23^	North American	0.9 %	N/A	N/A	N/A	N/A	N/A	N/A
Mashyakhy et al., 2019^6^	Saudi Arabian	7 %	21 %	14 %	DBR + PR	MBR + PR	8.3 %	32.1 %
Kim et al., 2012^24^	Korean	0.73 %	10.71 %	N/A	N/A	N/A	N/A	N/A
Marcano – Caldera et al., 2019^8^	Latin - American	23.6 %	57.6 %	43 %	DBR + PR	MBR + DBR + PR	N/A	N/A
Wu et al., 2017^22^	Chinese	N/A	31.91 % in males 48.44 % in females	N/A	N/A	MBR + PR in males MBR + DBR in females	N/A	N/A
Zheng et al., 2010^4^	Chinese	2.71 %	N/A	N/A	MBR + DBR	N/A	N/A	N/A
Allawi et al., 2024^3^	Syrian	N/A	7.6 %	N/A	N/A	N/A	N/A	N/A

MBR – Mesiobuccal root DBR – Distobuccal root PR – Palatal root.

MFM – Maxillary first molar.

MSM – Maxillary second molar.

Although root fusion in molars has moderate relevance to endodontics, it holds greater significance in oral surgery and periodontics. However, given that many fused roots present with merging main canals, this knowledge is highly pertinent to both root canal therapy and endodontic microsurgery. This study findings elucidate many aspects of root fusion in a Sri Lankan population, especially variations in prevalence with respect to sex and ethnicities. It also highlights that a strong clinical suspicion of root fusion in maxillary second molars should be maintained in patients with fused roots in maxillary first molars This underscores the need for advanced imaging techniques such CBCT as an adjunctive imaging modality and CBCT imaging (small field of view) can be considered when a preoperative periapical radiograph shows signs of fused rooted maxillary molars to diagnose and treat such teeth with predictable outcomes.

A significant limitation of the present study may be the low image resolution of the CBCT technology when compared with micro-CT which will limit the details of root canal morphology obtained by CBCTs. Therefore, in this study fine details of the root canal system such as apical deltas, isthmuses and extra root canals, such as the MB2, were not assessed. As micro – CT is currently unavailable in Sri Lanka, CBCT data was used in the present study which allowed us to perform an in vivo study on a large sample and make bilateral comparison and comparison between first and second maxillary molars on the same individual which would not be possible with micro – CT. Another limitation was the sample in the present study was a convenience sample in a single centre, therefore generalization about the total population must be made with due caution. Multicentre studies using random sampling methods needs to be conducted to provide a better overview about the population. Inclusion of patients with missing teeth and no information regarding their anatomy is also an additional limitation as one of the several possible reasons for these missing teeth may have been the complex anatomies (which may include some of the ones we were searching for) and subsequent failed root canal therapies.

## 6 Conclusions

Prevalence of root canal fusion was 5.1 % in maxillary first molars and 43.2 % in maxillary second molars. Commonest type of fusion was fusion of mesiobuccal and distobuccal roots in maxillary first molars and fusion of mesiobuccal and palatal roots in maxillary second molars and the relationship between the type of fusion and type of molar was statistically significant Root fusion was more prevalent among females. A high degree of suspicion for root fusion in maxillary second molars should be maintained in patients who exhibit root fusion in maxillary first molars. Presence of root canal merging in the fused root in maxillary first molar with root fusion was significantly higher than in literature therefore may pose additional challenges during endodontic therapy. The clinicians should be aware of unusual root and root canal configurations and should supplement the conventional radiographs with additional imaging when degree of clinical suspicion is high.

## Patient's/Guardian's consent

Not applicable.

## Ethical clearance

Ethics approval has been obtained from Ethics Review Committee, Faculty of Dental Sciences, University of Peradeniya, Sri Lanka – ERC/FDS/UOP/2022/17 dated October 18, 2022.

## Author contributions

All authors have accepted responsibility for the content of the manuscript, reviewed all results, and approved the final version.

Conceptualization R.D.J., M.C.N.F, R.M.J, R.D.W, data collection: R.D.W.; Statistical analysis: R.D.W; data interpretation: R.D.J, R.D.W; writing the first draft: R.D.W.; Manuscript editing and reviewing: R.D.J, R.M.J, M.C.N.F.

## Data availability statement

The data that support the findings of this study are available from the corresponding author, R.D.W, upon reasonable request.

## Financial support and sponsorship

No funding was received for this work.

## Declaration of competing interest

The authors declare no conflicts of interest.
